# Causal attributions of impulsive and compulsive behaviors

**DOI:** 10.3389/fpsyt.2024.1446972

**Published:** 2024-07-18

**Authors:** Karla Astudillo-Reyes, Ana I. Sánchez, María Luna-Adame, María Pilar Martínez, Lucas Muñoz-López

**Affiliations:** Department of Personality, Evaluation and Psychological Treatment, Faculty of Psychology, University of Granada, Granada, Spain

**Keywords:** impulsive, compulsive, drug addiction, alcohol, gender-based violence

## Abstract

**Introduction:**

Aggression, and therefore gender-based violence, can be an impulsive or compulsive behavior, depending on the consumption of alcohol and/or drugs. In Europe, the prevalence of gender-based violence is 16 to 23%. This prevalence shows that there is a need to make further progress in the treatment of aggression against women. Qualitative techniques allow us to understand perceptions and attributions holistically by analyzing what people who commit the crime say, why they say it and how they say it.

**Aim:**

To explore the experience of physical and verbal aggression by a partner, dependent on the presence or absence of alcohol and drug use, in the prison population.

**Method:**

A mixed methodology was used (combining qualitative and quantitative techniques). The sample was made up of 140 men divided into two focus groups [with alcohol and/or drug consumption (SAD) and without alcohol and/or drug consumption (NSAD)] who completed the Demographic, Criminal and Behavioral Interview in Penitentiary Institutions; the Gender Violence Questionnaire (both developed for this study) and the MultiCAGE CAD-4 Questionnaire. Qualitative data were analyzed using thematic analysis and quantitative data were obtained using contingency tables.

**Results:**

It was found that the SAD group attributed the crime committed to alcohol and/or drug consumption, while the NSAD group attributed it to jealousy and to their partner. The SAD group revealed that the consequence of the physical aggressions was to get what they were looking for from their partner and the consequences of the verbal aggressions was regret, unlike the NSAD group that did not get anything from the aggressions. The SAD group recognized that to avoid future aggressions they would have to avoid alcohol and/or drug use, while the NSAD group mentioned that they would have to avoid contact with their partner.

**Discussion:**

The need to include perceptions and attributions as well as the use of alcohol and/or drugs is emphasized when assessing individuals who commit the crime of gender-based violence.

## Introduction

Gender-based violence (GBV) is defined as a set of acts of physical and psychological violence, produced by aggressions to sexual freedom, threats, coercion and arbitrary deprivation of liberty. This type of violence also includes behaviors that reflect discrimination, inequality and power relations exercised by men against women, specifically by those who are or have been their spouses or who have maintained similar affective relationships, with or without cohabitation. Such acts may be manifested in both the public and private spheres (1). It is estimated that 1 in 3 women over the age of 15 has suffered GBV at least once in their life, making it a primary concern in terms of public health, gender equity, and human rights worldwide (2). In Europe, there is a 16% to 23% prevalence of GBV; specifically, 43% of psychological violence, 20% of physical violence, 12% of economic violence and 7% of sexual violence (3). In Spain, the European Survey on GBV revealed that 28.7% of women between the ages of 16 and 74 have experienced some form of violence by their partner throughout their lives (4). Consequently, 11.3% of the prison population is serving sentences for GBV crimes. The sentences are aimed at reeducation and social reintegration through specialized rehabilitation treatments. However, there is a recidivism rate of 41-60% for GBV crimes, making it one of the crimes with the highest recidivism rates in the country (5).

In classical research (6, 7), aggressive behavior has been linked to two subtypes: impulsive aggressive behavior and premeditated (compulsive) aggressive behavior. Impulsive aggression is defined as an aggressive response that arises in response to provocation and leads to a loss of behavioral control. On the other hand, premeditated (compulsive) aggression is a planned or conscious aggressive act that is not related to a state of agitation due to anger issues. Alcohol and drug use are considered impulsive behaviors (8–10), while GBV could be associated with compulsive behaviors (11). The association between aggression and impulsive or compulsive behaviors has been linked to inefficient frontal lobe function, reflecting complex neurocircuits (12). Although these terms are often used in clinical contexts, they are often imprecise and contradictory, necessitating further exploration of the topic. Conversely, the association between alcohol and drug use and GBV has been widely documented in quantitative research (13–16).

However, there is a need for qualitative research to analyze the factors influencing GBV as perceived by the perpetrators. Much of what we know about this topic comes from studies that have used quantitative measures to characterize an individual’s use of violent acts over a specific period (17). A recent study (18) highlights that these measures have been criticized for not considering the context in which aggression occurs. For example, physical aggression may differ in severity and meaning depending on the motive for the aggression and the cultural context in which it occurs. That is, the aggressive act can be perceived as memorable or distressing, depending on the conflict in which it occurs or the prior history of violence. Therefore, qualitative studies (19) allow for a holistic examination of contextual factors and the subjective meaning of violence. The words of the person who committed the crime when describing an aggressive act provide information on how they perceive that experience and what motivated them to do it. It also allows for understanding the links between events and emotions that drive criminal behavior.

The complexity of the explanatory variables of GBV has sparked particular interest in variables related to attributions (20, 21), motivations (18, 22, 23), and alcohol and/or drug use (17, 19, 24) among people who have committed GBV crimes and exhibit alcohol and/or drug use.

Regarding causal attributions, it has been found (20) that individuals who committed GBV crimes transferred responsibility for their behavior, especially to the victim. That is, they presented external attributions of guilt and minimized or denied the criminal behavior. It has also been found (21) that the causal explanations for GBV crimes were closely related to the expectations of the perpetrators (provoked by patriarchal views) about their partners’ behavior, lack of affection, poor communication, economic problems, and jealousy. Participants blamed their partners, denied responsibility for the crime, and attributed it to a lack of impulse control.

In terms of perceived motivations for criminal behavior, the results of a study (22) revealed that the aggressions committed in GBV crimes were learned behaviors from childhood in the family environment (participants had witnessed physical violence suffered by their mother, had been abused by their caregivers, and later exercised violence against their children and partners). This behavior pattern reveals the transmission of violence from generation to generation, becoming a normalized behavior and one of the main motivations for the crime. Additionally, participants mentioned that they assaulted the victim as a result of relationship problems characterized by jealousy, revenge, ingratitude, and sadness. Finally, it is noted that those who committed the crime perceived themselves as victims of the judicial system because they considered the complaint and consequences to be unjust, provoking desires for revenge against their partner.

Likewise, the motivational factors for committing the GBV crime in people who were receiving treatment for having assaulted their partner have been analyzed (23). The thematic analysis found that the factors motivating the commission of a GBV crime were adverse childhood experiences (bullying, neglect in upbringing, physical or sexual violence), communication problems with the partner (arguments, lack of mutual listening, and denial of the existence of problems), the outcome obtained as a result of the aggression (information, causing harm, revenge), and the positive interpretation of the consequences of the aggression (achieving their goal and continuing the relationship after the aggression). Similarly, the reasons for the use of physical aggression by people who have committed GBV crimes were studied (18) and found that there were three main reasons. The first reason for the use of physical aggression was to express emotions and feelings. Participants described physical aggression as something that allowed them to express their discomfort and disagreement with their partner when verbal expression was inadequate. The second reason for the use of physical aggression was instrumental, meaning they assaulted their partner to achieve a specific purpose (to distance their partner to end the conflict or to detain their partner to continue the conflict). Lastly, the third reason for the use of physical aggression was punishment. Participants mentioned that they assaulted their partner to punish them for infidelity, for assaulting them, or for the victim’s drug use.

Finally, regarding the influence of alcohol and/or drug use on GBV, a study (24) analyzed the behaviors, interactions, and conditions that occurred before, during, and after GBV, according to the perspective of those who committed the crime. It was found that before the violent act, there were feelings of contempt towards the victim due to relationship conflicts provoked by the victim’s recurrent threats to leave or take their children and refusal to have sexual relations. Additionally, there were communication problems, economic difficulties, work stress, and alcohol and/or drug use by the person who committed the crime. During the violent act, participants highlighted those feelings of anger and frustration, and the use of alcohol and/or drugs triggered the GBV crime (shouts, insults, and hitting). Finally, it was found that after the violent act, those who committed a GBV crime exhibited feelings of guilt, remorse, and behaviors such as distancing, reconciliation, and alcohol and/or drug use. Lastly, it was found that participants tried to prevent violent incidents at all stages (before, during, and after). To achieve this, they avoided talking about conflicting topics with their partner, vented with friends and family, and went out to consume alcohol and/or drugs. It has also been evidenced (19) that those who committed GBV crimes justified their criminal behavior with the effect of alcohol and/or drug use or abstinence and the stress they felt due to relationship conflicts (jealousy, suspicion of infidelity, breakups), unemployment, and economic problems. Justifying criminal behavior is a commonly used mechanism by those who commit GBV crimes to give moral sense to violent behaviors, thus alleviating feelings of guilt and avoiding social exclusion. This aspect was also evidenced in a qualitative study (17) in which it was found that participants perceived that the crime committed was solely due to alcohol and/or drug addiction (both under its effects and under the effects of withdrawal syndrome) and showed a minimization of criminal behavior, indicating that GBV incidents were isolated and unusual, caused by the loss of control due to jealousy. Conversely, their partners or ex-partners described the GBV incidents as continuous and highly dangerous, not isolated and unusual events.

Studies focused on attributions, motivations, and alcohol and/or drug use related to GBV reveal that those who commit these crimes do not accept responsibility for their behavior and minimize the consequences of violence. They present attributions characterized by the denial of personal responsibility, blaming the victim, and other external attributions of guilt (family problems, effects of alcohol and/or drugs, economic difficulties) that allow them to justify their criminal conduct. Therefore, increasing our understanding of why GBV occurs from the perspective of the perpetrator is essential for developing effective treatments. This aspect is even more important given the poor effectiveness of treatments aimed at this population (25, 26), due to limitations in studies focused on this topic. Specifically, the limitations of studies on GBV relate, firstly, to the excessive use of quantitative methodologies (13). Secondly, the few qualitative studies conducted with those who have committed GBV crimes have been carried out with unrepresentative samples, preventing the generalization of the results (20, 27). Thirdly, the perceptions of this population regarding the type of violence exerted, for example, physical or verbal, have not been analyzed (18). Lastly, it has not been studied whether people who consume alcohol and/or drugs present causal attributions for the crime differently from those presented by people who do not consume alcohol and/or drugs (28). For this reason, the objective of this study is to explore the experiences related to physical and verbal partner aggression, based on the presence or absence of alcohol and drug use, in a prison population.

## Materials and methods

### Participants

The sample consisted of 140 men, with a mean age of 40.08 years (SD = 10,85), selected through intentional sampling at the Penitentiary Center of Granada (Spain). The only prison treatment they were receiving at the time of participating in this study was the intervention program aimed at people who commit crimes of gender violence. Sampling was carried out during the first two weeks of said treatment. Participants were divided into two focal groups based on the presence or absence of alcohol and/or drug consumption, according to the MultiCAGE CAD-4 (29). Group 1, with alcohol and/or drug consumption (SAD), comprised 70 men, with a mean age of 40.41 years (SD = 10,64). Group 2, without alcohol and/or drug consumption (NSAD), consisted of 70 men, with a mean age of 39.74 years (SD = 11,11). Inclusion criteria were being male, aged between 18 and 63 years, having committed a Domestic Violence and Gender Violence (GBV) offense, and agreeing to voluntary participation in the study by signing an informed consent form. Exclusion criteria were being over 63 years old, suffering from a physical or psychiatric illness (schizophrenia and/or depression), and currently undergoing psychopharmacological treatment. **Table 1** presents the sociodemographic characteristics of the described sample.

**Table 1:** Sociodemographic variables.

	Group with alcohol and/or drug consumption (SAD)	Group without alcohol and/or drug consumption (NSAD)	χ^2^/F	*p*
**Age (X/DT)**	40,41 (10,64)	39,74 (11,11)	0,133	0,716
**Civil Status (N)**			1,301	0,729
Single	34	31		
Married	18	15		
Divorced	11	14		
Domestic partner	7	10		
**Educational level (N)**			6,595	0,086
No education	6	5		
Primary/ESO	39	49		
Baccalaureate/HVTC	15	14		
Undergraduate/Postgraduate	10	2		
**Type of Crime (N)**			2,029	0,154
Injury crime	20	28		
Crime of threats	50	42		
**Penalty time (N)**			1,343	0,854
20 days - 1,22 months	20	20		
2 - 5 months	8	9		
6 - 9, 01 months	35	30		
10 - 16,04 months	4	6		
21-24 months	3	5		
**Penalty type (N)**			0,034	0,853
Work for the benefit of the community	20	21		
Prison	50	49		

### Instruments

The assessment instruments used in the present study were as follows:

*Demographic, Offenses, and Behaviors Interview in Penitentiary Institutions:* This interview was designed specifically for this study to collect sociodemographic data, type of offense, and participants’ sentence lengths and types.

*Gender Violence Questionnaire:* This questionnaire, developed for this study, aims to explore experiences related to physical and/or verbal partner aggression. It consists of 14 open-ended questions about events before, during, and after violent incidents (arguments, insults, assaults, and hits) and how such events could have been avoided. Completing this questionnaire takes 45 minutes, and the questions are based on the proposal by Ager (24).

*MultiCAGE CAD-4 Questionnaire* (29): This test evaluates the presence of addictive behaviors. It is self-administered and answered using a dichotomous scale (Yes/No). It consists of 32 items divided into 8 categories (alcohol, gambling, drugs, food, internet, video games, shopping, and sex). Each category contains 4 items related to 4 symptoms. Two affirmative responses indicate the possible existence of that problem, three affirmative responses suggest the highly likely existence of that problem, and four affirmative responses confirm the existence of that problem. It is a tool with high reliability (Cronbach’s alpha 0.86) and adequate criterion validity (between 90% and 100%).

### Procedure

The Demographic, Offenses, and Behaviors Interview in Penitentiary Institutions was conducted individually to verify the inclusion criteria and propose voluntary participation in this study. Participants were informed of their right to interrupt the procedure at any time, and their written consent was obtained. Additionally, they completed the MultiCAGE CAD-4 Questionnaire (29) to form the study groups (SAD and NSAD). The criterion for determining Group 1 (SAD) was to respond affirmatively to two or more questions related to alcohol and drug use in the MultiCAGE CAD-4 (29). In contrast, the criterion for determining Group 2 (NSAD) was to respond negatively to all questions or to respond positively to only one question related to alcohol and drug use in the MultiCAGE CAD-4 (29). Subsequently, participants autonomously completed the Gender Violence Questionnaire in groups to gather their main perceptions and attributions regarding relationship problems. Finally, the instruments were scored, and the data were interpreted and analyzed. Permission for this study was obtained from the Ethics Committee of the University of Granada (2254/CEIH/2021).

### Data analysis

The qualitative data were studied through a thematic analysis (30), which was carried out in six phases. The first phase was the familiarization with the data, in this initial phase several readings were made of the answers given by the participants in the qualitative questionnaire on gender-based violence, in order to identify possible patterns or emerging themes. The second phase was the generation of provisional codes, this phase consisted of giving a name (code) to the potentially relevant and common data mentioned by the participants. For example, in question 1, where they were asked to describe the event that provoked them to go to prison, it was observed that the participants presented common patterns in their answers, mentioning events provoked by *alcohol/drug use, jealousy, aggression or economic problems*. For this reason, these were the first codes assigned in this question. The third phase was the search for themes and sub-themes; in this phase, broader names were assigned that grouped the codes established in the previous phase. For example, in question 1, the codes *alcohol/drug use, jealousy, aggression and economic problems* were grouped into three subthemes (*self, partner, both*), which in turn were part of the theme *events*. The fourth and fifth phases were the review of themes and subthemes, in these phases the coherence and relevance of each of them was analyzed. For example, the need was identified to assign two new codes (*accepts aggression and does not accept aggression*) that belonged to the first subtheme (*self*) to give greater meaning to the participants’ responses. Finally, the sixth phase was the description of the results; this phase focused on making sense of all the themes, subthemes and codes identified in the previous phases in order to respond to the objective of the study. For example, in question 1, in this sixth phase, it was determined that participants attributed the blame for their crime primarily to events caused by their own alcohol/drug use and to the jealousy they felt for their partner. In addition, it was identified that they accepted the aggressions, minimizing the consequences of the events or denied the aggressions, justifying their behavior. Secondly, they attributed the blame for their crime to events provoked by their partner, as a consequence of alcohol/drug consumption, jealousy and aggression that they exercised against the participants, which caused them to physically or verbally assault them. Finally, in third place, they attributed the blame for their crime to events provoked by alcohol/drug consumption, jealousy and economic problems of both, which generated more couple conflicts.

The quantitative data were analyzed using the statistical program SPSS 26. First, to determine the sociodemographic characteristics of the sample, a descriptive statistical analysis was conducted. Secondly, contingency tables were created to demonstrate the differences between the groups (SAD and NSAD) according to the themes identified in the questionnaires.

## Results

As seen in **Table 2**, 14 themes were identified through thematic analysis. These themes were divided into 54 subthemes related to events (guilt as an attribution of aggression, partner, both); Feelings and behaviors following a couple’s problem (feelings, behaviors, nothing); Attributions of discussions (self, partner, both, no one); Attributions of aggressions (self, partner, both, no one); Behaviors at the end of a discussion (avoidance, resolving problems, continuing the discussion, nothing); Behaviors at the end of aggression (avoidance, resolving problems, continuing the discussion, nothing); Consequences of discussions (losing, getting what they wanted, regret, nothing); Consequences of aggressions (losing, getting what they wanted, regret, nothing); Reasons for discussions (self, partner, both, nothing); Reasons for aggressions (self, partner, both, nothing); Prevention of discussions (avoidance, self-control, ending the relationship, nothing); Prevention of aggressions (avoidance, self-control, ending the relationship, nothing); Prevention of future discussions (avoidance, self-control, ending the relationship, nothing); and Prevention of future aggressions (avoidance, self-control, ending the relationship, nothing).

**Table 2:** Categorization of open-ended responses on gender violence.

Themes	Subthemes	Description
1. Events	Guilt as an attribution of aggression (self)	Alcohol and/or drugs
		Jealousy
		Accepting the aggression
		Not accepting the aggression
	Partner	Alcohol and/or drugs
		Jealousy
		Aggression
	Both	Alcohol and/or drugs
		Jealousy
		Economic problems
2. Feelings and behaviors following a couple’s problem	Feelings	I feel bad, sad, frustrated, worried, guilty, regretful, helplessness
		Anger, anxiety
	Behaviors	Walking, walking away, ignoring her in solitude.
		Going out with friends, family.
		Talk, solve problems, ask for forgiveness.
		Use of alcohol, tobacco, drugs.
	Nothing	
3. Attributions of discussions	Self	
	Partner	
	Both	
	No one	
4. Attributions of aggressions	Self	
	Partner	
	Both	
	No one	
5. Behaviors at the end of a discussion	Avoidance	Going out to see friends, going for a walk, getting away
		Ignoring the partner
	Resolving problems	Talk to fix the problem, come to your senses
		Ask for forgiveness, reconcile, sex
	Continuing the discussion	Keep going until someone stops, stay angry, revenge
		Ending the relationship, prison
	Nothing	
6. Behaviors at the end of aggression	Avoidance	Going out to see friends, going for a walk, getting away
		Ignoring the partner
	Resolving problems	Talk to fix the problem, come to your senses
		Ask for forgiveness, reconcile, sex
	Continuing the discussion	Keep going until someone stops, stay angry, revenge
		Ending the relationship, prison
	Nothing	
7. Consequences of discussions	Losing	End the relationship, the situation gets worse, she gets angrier
		Complaint, prison, children, work
	Getting what they wanted	Understanding, being listened to, being silenced
		Venting anger, letting off steam, speaking my mind
		Revenge, leave me alone, defend me
	Regret	Feeling bad, frustration, low self-esteem, discomfort
	Nothing	
8. Consequences of aggressions	Losing	End the relationship, the situation gets worse, she gets angrier
		Complaint, prison, children, work
	Getting what they wanted	Understanding, being listened to, being silenced
		Venting anger, letting off steam, speaking my mind
		Revenge, leave me alone, defend me
	Regret	Feeling bad, frustration, low self-esteem, discomfort
	Nothing	
9. Reasons for discussions	Self	Alcohol/Drugs
		Jealousy
		Impulse, stress, explodes, revenge, rage, punishment, hurt
	Partner	Alcohol/Drugs
		Jealousy
		Provocations, insults first, abuses, does not listen to me, prevents visits to his children, does not fulfill obligations
	Both	Alcohol/Drugs
		Jealousy
		Lack of respect, toxic relationship, normal behavior, disagreements
	No one	
10. Reasons for aggressions	Self	Alcohol/Drugs
		Jealousy
		Impulse, stress, explodes, revenge, rage, punishment, hurt
	Partner	Alcohol/Drugs
		Jealousy
		Provocations, insults first, abuses, does not listen to me, prevents visits to his children, does not fulfill obligations
	Both	Alcohol/Drugs
		Jealousy
		Lack of respect, toxic relationship, normal behavior, disagreements
	No one	
11. Prevention of discussions	Avoidance	Stop consuming alcohol/drugs
		Walking away, going for a smoke, staying at work, staying quiet, staying still, sticking objects
	Self-control	Controlling my emotions, calming myself, breathing, counting to 10, crying, biting my tongue
		Psychological therapy
		Thinking about the consequences, reflecting, putting myself in their shoes, empathizing
		Resolve conflict, talk respectfully, converse, give gifts, have sex, etc.
	Ending the relationship	Separate, do not continue, end at the first sign
	Nothing	
12. Prevention of aggressions	Avoidance	Stop consuming alcohol/drugs
		Walking away, going for a smoke, staying at work, staying quiet, staying still, sticking objects
	Self-control	Controlling my emotions, calming myself, breathing, counting to 10, crying, biting my tongue
		Psychological therapy
		Thinking about the consequences, reflecting, putting myself in their shoes, empathizing
		Resolve conflict, talk respectfully, converse, give gifts, have sex, etc.
	Ending the relationship	Separate, do not continue, end at the first sign
	Nothing	
13. Prevention of future discussions	Avoidance	Stop consuming alcohol/drugs
		Walking away, going for a smoke, staying at work, staying quiet, staying still, sticking objects
	Self-control	Controlling my emotions, calming myself, breathing, counting to 10, crying, biting my tongue
		Psychological therapy
		To think about the consequences, to reflect, to put myself in their shoes, to empathize
		Resolve conflict, talk respectfully, converse, give gifts, have sex, etc.
	Ending the relationship	Separate, do not continue, end at the first sign
	Nothing	
14. Prevention of future aggressions	Avoidance	Stop consuming alcohol/drugs
		Walking away, going for a smoke, staying at work, staying quiet, staying still, sticking objects
	Self-control	Controlling my emotions, calming myself, breathing, counting to 10, crying, biting my tongue
		Psychological therapy
		To think about the consequences, to reflect, to put myself in their shoes, to empathize
		Resolve conflict, talk respectfully, converse, give gifts, have sex, etc.
	Ending the relationship	Separate, do not continue, end at the first sign
	Nothing	

We found statistically significant differences between the groups (SAD and NSAD) in five themes identified in the questionnaires (**Table 3**). The first theme, “Event: guilt as an attribution of aggression” (χ2 = 12.518; p=0.014) had the highest positive frequencies for alcohol and/or drugs in the SAD Group; and jealousy, accepting the aggression, and not accepting the aggression were highest in the NSAD Group. The second theme, “Consequences of discussions: regret” (χ2 = 4.155; p = 0.042) had the highest positive frequencies of feeling bad, frustration, low self-esteem, and discomfort in the SAD Group. The third theme, “Consequences of aggressions: getting what they wanted” (χ2 = 11.082; p=0.011) had the highest positive frequencies for understanding, being listened to, being silenced; venting anger, catharsis, saying what I think; revenge, being left in peace, and defending oneself, in the SAD Group. The fourth theme “Consequences of aggressions: nothing” (χ2 = 11.459; p = 0.001) had the highest positive frequencies in the NSAD Group. The fifth theme, “Prevention of future discussions: avoidance” had the highest positive frequencies for stopping alcohol and/or drug use in the SAD Group; and moving away, going to smoke, staying at work, staying silent, still, and hitting objects in the NSAD Group.

**Table 3:** Differences in perceptions and attributions of the crime GBV based on the presence or absence of alcohol and/or drug consumption.

Themes	Subthemes	Group with alcohol and/or drug consumption (SAD)	Group without alcohol and/or drug consumption (NSAD)	χ^2^	*p*
**Event 1** **(Guilt as an attribution of aggression - self)**				**12,518**	**0,014**
	Another answer	25 (55,6%)	20 (44,4%)		
	Alcohol and/or drugs	9 (100%)	0 (0%)		
	Jealousy	6 (46,2%)	7 (53,8%)		
	Accepting the aggression	3 (30%)	7 (70%)		
	Not accepting the aggression	27 (42,9%)	36 (57,1%)		
**Event 2 (Partner)**				2,095	0,553
	Another answer	57 (50,4%)	56 (49,6%)		
	Alcohol and/or drugs	57 (50,4%)	56 (49,6%)		
	Jealousy	9 (60%)	6 (40%)		
	Aggression	2 (28,6%)	5 (71,4%)		
**Event 3 (Both)**				6,083	0,103
	Another answer	58 (47,5%)	64 (52,5%)		
	Alcohol and/or drugs	2 (66,7%)	1 (33,3%)		
	Jealousy	9 (81,8%)	2 (18,2%)		
	Economic problems	1 (25%)	3 (75%)		
**Consequences of discussions 1 (Losing)**				1,144	0,564
	Another answer	49 (53,3%)	43 (46,7%)		
	End the relationship, the situation gets worse, she gets angrier	18 (43,9%)	23 (56,1%)		
	Complaint, prison, children, work	3 (42,9%)	4 (57,1%)		
**Consequences of discussions 2 (Getting what they wanted)**				1,923	0,589
	Another answer	47 (48,5%)	50 (51,5%)		
	Understanding, being listened to, being silenced	9 (47,4%)	10 (52,6%)		
	Venting anger, letting off steam, speaking my mind	10 (66,7%)	5 (33,3%)		
	Revenge, leave me alone, defend me	4 (44,4%)	5 (55,6%)		
**Consequences of discussions 3 (Regret)**				**4,155**	**0,042**
	Another answer	60 (47,2%)	67 (52,8%)		
	Feeling bad, frustration, low self-esteem, discomfort	10 (76,9%)	3 (23,1%)		
**Consequences of discussions 4 (Nothing)**				0,598	0,439
	Another answer	54 (51,9%)	50 (48,1%)		
	Nothing	16 (44,4%)	20 (55,6%)		
**Consequences of aggressions 1 (Losing)**				1,008	0,604
	Another answer	65 (49,6%)	66 (50,4%)		
	End the relationship, the situation gets worse, she gets angrier	4 (50%)	4 (50%)		
	Complaint, prison, children, work	1 (100%)	0 (0%)		
**Consequences of aggressions 2 (Getting what they wanted)**				**11,082**	**0,011**
	Another answer	54 (44,6%)	67 (55,4%)		
	Understanding, being listened to, being silenced	5 (71,4%)	2 (28,6%)		
	Venting anger, letting off steam, speaking my mind	2 (100%)	0 (0%)		
	Revenge, leave me alone, defend me	90 (90%)	1 (10%)		
**Consequences of aggressions 3 (Regret)**				1,867	0,172
	Another answer	66 (48,9%)	69 (51,1%)		
	Feeling bad, frustration, low self-esteem, discomfort	4 (80%)	1 (20%)		
**Consequences of aggressions 4 (Nothing)**				**11,459**	**0,001**
	Another answer	25 (75,8%)	8 (24,2%)		
	Nothing	45 (42,1%)	62 (57,9%)		
**Prevention of future discussions 1 (Avoidance)**				**7,085**	**0,029**
	Another answer	55 (53,9%)	47 (46,1%)		
	Stop consuming alcohol/drugs	3 (100%)	0 (0%)		
	Walking away, going for a smoke, staying at work, staying quiet, staying still, sticking objects	12 (34,3%)	23 (65,7%)		
**Prevention of future discussions 2 (Self-control)**				3,528	0,474
	Another answer	31 (43,1%)	41 (56,9%)		
	Controlling my emotions, calming myself, breathing, counting to 10, crying, biting my tongue	12 (54,5%)	10 (45,5%)		
	Psychological therapy	2 (66,7%)	1 (33,3%)		
	To think about the consequences, to reflect, to put myself in their shoes, to empathize	8 (66,7%)	4 (33,3%)		
	Resolve conflict, talk respectfully, converse, give gifts, have sex, etc.	17 (54,8%)	14 (45,2%)		
**Prevention of future discussions 3 (Ending the relationship)**				2,120	0,145
	Another answer	61 (48%)	66 (42%)		
	Separate, do not continue, end at the first sign	9 (69,2%)	4 (30,8%)		
**Prevention of future discussions 4 (Nothing)**				2,745	0,098
	Another answer	63 (52,9%)	56 (47,1%)		
	Nothing	7 (33,3%)	14 (66,7%)		

Significant differences p=<0.05 are highlighted.

## Discussion

In this study, the experiences related to physical and verbal partner aggression were explored based on the presence or absence of alcohol and drug use in the prison population. To achieve this objective, a mixed methodology was used, which consists of combining qualitative and quantitative techniques, allowing for a deep understanding of the phenomenon under study (31). The results revealed that there are statistically significant differences in the experiences related to physical and verbal partner aggression between the study groups (SAD and NSAD). In other words, the presence or absence of alcohol and/or drug use in individuals who commit gender violence (GBV) offenses influences how they perceive their reality and how they manifest aggressive behavior (impulsive or compulsive).

Specifically, there were differences between the groups regarding the perception of “guilt as an attribution of aggression”. The SAD group mentioned more frequently than the NSAD group that they were the ones who caused the events that led to their imprisonment as a consequence of their alcohol and/or drug use. Participants indicated that their “use of alcohol and/or drugs” was the main trigger for the violent event, attributing their behavior to the substance’s effect. This result is consistent with previous studies (19, 27) that reveal men who assault their partners consider alcohol and/or drugs a stress factor that provokes their offense. However, it has been observed that this is a way to justify their lack of self-control and neutralize their responsibility for the acts in order to maintain a positive self-identity (19). This result is also reaffirmed by various authors (13–17) who have demonstrated a close relationship between alcohol and/or drug use and GBV.

In relation to the NSAD group, three types of attributions (jealousy, external, and internal) related to aggression were more frequently found compared to the SAD group. Regarding “jealousy” as an attribution for aggression, participants mentioned they assaulted their partner due to distrust and fear of being betrayed. This result is consistent with findings in various studies (20, 32, 33), which also identified jealousy as one of the main causes of GBV. Specifically (32), it has been demonstrated that participants who commit this offense make causal attributions, such as expressions of anger at disagreement or betrayal by the partner. Additionally (20), found that jealousy arises from a need for dominance and exclusivity, manifested in supervision and coercive control behaviors over the woman’s autonomy. Concerning external attributions of aggression, we observed that the NSAD group “does not accept the aggression” more frequently than the SAD group. Participants stated they did not assault their partner and that during the trial, they only accepted the aggression based on legal advice to reduce their sentence. This finding is coherent with other studies (17, 21), which found that denying responsibility for the offense is a commonly used mechanism for addressing conflicts. Finally, regarding internal attributions of aggression, we found that the NSAD group “accepts the aggression” more frequently than the SAD group. In this case, participants acknowledged assaulting their partner but indicated that their violent behavior occurred as a normal reaction during an argument where they could not control their anger. This result aligns with a previous study (28), which identified that individuals who commit a GBV offense tend to minimize the consequences of their acts and justify their behavior when acknowledging the aggression. This minimization is due to the normalization of violence use and masculinity stereotypes.

We also found differences between the study groups (SAD and NSAD) regarding the “consequences of physical and verbal aggression”. Participants mentioned that physical aggression manifested through hitting, pushing, or slapping. Verbal aggression occurred during arguments with insults, shouting, or threats. For “physical aggression”, the SAD group more frequently indicated that the consequence of physically assaulting their partner was “getting what they wanted,” unlike the NSAD. That is, through aggression, they made their partner listen, retaliated, and vented their anger. This result is consistent with literature (16, 18) highlighting various functions related to using physical aggression towards a partner. Among the most common functions, aggression is used as an instrument to get what they want from their partner, as revenge for the partner’s behavior, and as an emotional outlet (18). It has also been observed that there is physical aggression in GBV (16).

Regarding perceived consequences of “physical aggression”, we also found that the NSAD group mentioned more frequently than the SAD group that physical aggression “did not get what they wanted” from their partner. Participants indicated that aggression did not help them achieve their desired outcome. On the contrary, after physically assaulting their partner, they had more problems (legal, family, and social). This result also highlights the important role of alcohol and/or drugs in achieving what they wanted from their partner. Participants who used alcohol and/or drugs got what they wanted more frequently than those who did not use substances. This result is consistent with other authors’ findings (22), emphasizing the relationship between increasing or decreasing criminal behaviors (problematic alcohol and/or drug use and GBV) and the type of consequences for those who commit these offenses.

On the other hand, regarding the results on the consequences of “verbal aggression,” the SAD group more frequently expressed “regret” compared to the NSAD group. Participants described regret as a feeling of discomfort, sadness, and frustration after assaulting their partner. This result aligns with recent studies (23, 24), which found that the discomfort caused by aggression generates a need to remedy the damage through promises of change and reconciliation attempts. This result has significant clinical importance in treating individuals who commit GBV offenses and consume alcohol and/or drugs. Regret can provoke greater reflection on the acts, becoming an opportunity to generate awareness of personal responsibility for self-behavior.

Finally, we found differences between the groups (SAD and NSAD) regarding the “prevention of future verbal aggression”. Specifically, the SAD group more frequently mentioned that the strategy for preventing future verbal aggression would be “abstaining from alcohol and/or drugs”. In contrast, the NSAD group more frequently mentioned that the best way to prevent verbal aggression would be “avoiding contact”. For the SAD group, participants who attributed their behavior solely to alcohol and/or drugs considered abstinence the best solution. This result is consistent with various authors’ findings (13, 14) who found a direct relationship between alcohol and/or drug use and the recurrence of GBV offenses. For the NSAD group, participants mentioned that to avoid verbally assaulting their partner in the future, they would resort to behaviors that avoid confrontation, such as staying silent, distancing themselves, or going out for a cigarette. These prevention strategies were also observed in a previous study (24), highlighting that individuals who commit a GBV offense try to prevent aggression before, during, and after a violent event. Therefore, it emphasizes the need to focus treatment on strengthening these prevention strategies through emotion management and cognitive restructuring to generate more stable behavior changes.

This study allows us to draw three crucial conclusions about the differences between the study groups (SAD and NSAD) regarding experiences related to physical and verbal partner aggression. First, regarding “guilt as an attribution of aggression”, we found that the SAD group attributes aggression towards the partner solely to alcohol and/or drug use, while the NSAD group more frequently attributes it to jealousy. Additionally, this group presents an external attribution when not accepting the aggression and an internal attribution when accepting the aggression, although minimizing responsibility. Second, regarding the “consequences of physical and verbal aggression”, we found that for physical aggression, the SAD group mentioned that the consequence of physically assaulting their partner was getting what they wanted (being heard, revenge, and expressing anger), unlike the NSAD group, who did not get what they wanted outcome with physical aggression. For verbal aggression consequences, the SAD group more frequently expressed regret compared to the NSAD group. Finally, regarding the “prevention of future verbal aggression”, the SAD group more frequently mentioned that abstaining from alcohol and/or drugs would be the best decision to avoid verbally assaulting their partner, in contrast to the NSAD group, who more frequently suggested avoiding contact with their partner.

It is necessary to mention that this study has three limitations. The first limitation is that the sample was composed only of men, as it only studied individuals who had committed GBV offenses. However, to deepen knowledge about intimate partner aggression, the perceptions of women who have committed or received violence should also be evaluated. The second limitation is the absence of a control group, meaning we did not study individuals who had not been convicted of GBV offenses. Therefore, it is recommended that this study be replicated with a non-prison sample. Finally, the third limitation is the bias in participants’ responses. Biases can occur unconsciously (due to memory errors related to past events) or consciously (due to social desirability). Nevertheless, it is also important to highlight three significant strengths. The first strength is the use of a mixed methodology, which has allowed us to leverage the richness of qualitative and quantitative techniques to deepen our understanding of GBV. Qualitative techniques enabled a holistic understanding of the perceptions and attributions of GBV from the perspective of the perpetrator. Quantitative techniques allowed us to know the frequency and statistical differences of these perceptions between study groups (SAD and NSAD). The second strength is that the results of this study present high reliability and applicability, especially due to data saturation and the methodological triangulation used in the analysis. Lastly, the third strength is that it is the study with the largest number of participants (N = 140) that analyzes both qualitatively and quantitatively two of the most common issues in the prison population and most relevant to public health in Spain (alcohol and/or drug use and GBV).

The results of this study have important practical implications, especially in the treatment of people who commit GBV offenses. Knowing the perceptions and attributions of the crime committed, as well as the role of alcohol and/or drug use in partner aggression, is essential to identify the cognitive distortions that maintain this behavior. In other words, these results help us to increase the specificity of treatments, which, in turn, enhance adherence to therapy, motivation to change and prevention of recidivism. Specifically, the repentance shown by the participants as a consequence of the crime can be a key tool to promote awareness of their actions and encourage significant behavioral changes. In addition, knowing the prevention strategies used by the participants (avoiding alcohol and/or drug use and contact with their partner during a conflict) allows us to strengthen these strategies in intervention treatments, through specific components aimed at alcohol and/or drug use cessation, conflict resolution, emotion management and cognitive restructuring. Finally, we recommend that future lines of research focus on conducting comparative studies to learn about the experiences of physical and verbal partner aggression among subgroups. For example, we could compare people with different types of drug use or criminal records. This would help to design more personalized treatments.

## Data availability statement

The raw data supporting the conclusions of this article will be made available by the authors, without undue reservation.

## Ethics statement

The studies involving humans were approved by Ethics Committee of the University of Granada (2254/CEIH/2021). The studies were conducted in accordance with the local legislation and institutional requirements. The participants provided their written informed consent to participate in this study.

## Author contributions

KA-R: Investigation, Supervision, Writing – original draft. AIS: Methodology, Supervision, Writing – original draft. ML-A: Data curation, Formal analysis, Investigation, Validation, Writing – review & editing. MPM: Formal analysis, Investigation, Supervision, Writing – review & editing. LM-L: Conceptualization, Investigation, Methodology, Writing – review & editing.
